# Enabling the analysis of patient-level data across jurisdictions for research use: a real-world exploration of a federated likelihood approach

**DOI:** 10.23889/ijpds.v11i1.3160

**Published:** 2026-04-28

**Authors:** Megan Harmon, Na Li, Tolulope Sajobi, Jessalyn Holodinsky, Tyler Williamson

**Affiliations:** 1 Department of Community Health Sciences, Cumming School of Medicine, University of Calgary, Calgary, Alberta, Canada; 2 Centre for Health Informatics, University of Calgary, Calgary, Alberta, Canada; 3 Department of Emergency Medicine, University of Calgary, Calgary, Alberta, Canada; 4 Department of Clinical Neurosciences, University of Calgary, Calgary, Alberta, Canada; 5 O’Brien Institute for Public Health, University of Calgary, Calgary, Alberta, Canada; 6 Hotchkiss Brain Institute, University of Calgary, Calgary, Alberta, Canada; 7 Alberta Children’s Hospital Research Institute, University of Calgary, Calgary, Albera, Canada

**Keywords:** likelihood functions, meta-analysis, federated analysis

## Abstract

**Introduction:**

The disclosure of health data is governed by strict privacy regulations which significantly restrict the transfer of data across jurisdictions. These limitations restrict the scope of health research, particularly the ability to conduct studies that span multiple jurisdictions (e.g. provinces, states, or countries). One common practice is to use meta-analysis to pool jurisdiction-specific estimates. However, this approach relies on combining aggregate-level data, which may overlook important nuances. Therefore, alternative methods are needed. This study investigates the potential of a federated likelihood approach to analyse health data across jurisdictions while keeping the data inside the jurisdiction.

**Objectives:**

(1) Evaluate the effectiveness of the meta-analysis technique in pooling jurisdiction-specific results to produce overall estimates (2) assess the utility of a federated likelihood approach for interjurisdictional health data analysis; and (3) compare the performance of a federated likelihood method with the conventional meta-analysis approach.

**Methods:**

Log-likelihood functions from separate jurisdictions were directly combined to form a single overall log-likelihood function, and overall parameter estimates. We show that these parameter estimates are mathematically equivalent to those obtained from analysing the pooled individual-level data. Using data from the Canadian Primary Care Sentinel Surveillance Network, and using chronic kidney disease as a case study, estimates from five jurisdictions are combined using meta-analysis and the federated likelihood approach. These results are compared to the overall estimates from a global model.

**Result:**

The likelihood method demonstrated strong performance in analysing federated data, yielding the lowest relative percent absolute bias at 1.34%. This was notably lower than the bias observed with the common effect meta-analysis (4.09%).

**Conclusion:**

The federated likelihood method offers a promising approach to interjurisdictional health data analysis. However, further investigation is needed to assess its feasibility across different model types and model complexity to fully understand the scope of its utility.

## Introduction

As health systems rely on data to drive decision-making and improve outcomes, the ability to analyse health data distributed across multiple institutions and regions has become essential [1, 2]. While some research questions can be addressed within a single organisation, many critical public health and clinical challenges – such as understanding rare events or chronic disease surveillance – demand a broader, more integrated approach [3–5]. In such cases, no single data source may offer sufficient scale or diversity to produce reliable insights. Federated health data analysis allows researchers to overcome these limitations by synthesising the statistical structure of the data across locations [6], thereby enabling more accurate estimates, detection of rare outcomes, and data-driven public health policies that are both comprehensive and generalisable. Without such collaboration, key patterns and risks may remain undetected, limiting the effectiveness of health interventions and decision-making at scale.

Globally, the disclosure of health data is subject to specific jurisdictional regulations. Generally, these regulations, whether they are organisational policies or government legislation, restrict the disclosure, transfer, and sharing of patient-level data across regional boundaries, particularly for research use. Countries around the world face barriers when conducting cross-jurisdictional health research due to varying privacy regulations. In Canada, provincial legislation like the Alberta Health Information Act and the Ontario Personal Health Information Protection Act governs the collection, use, and disclosure of health information [7, 8]. The European Union’s General Data Protection Regulation (GDPR) imposes strict limits on the sharing of personal health data between member states [9]. Similarly, in the United States, the Health Insurance Portability and Accountability Act (HIPAA) enforces privacy standards that can make cross-state health data sharing difficult [10].

As cross-border collaboration and data-driven health research become increasingly important, finding secure, ethical, and accurate methods for analysing federated health data remains a pressing international challenge.

Meta-analysis is a well-established method for synthesising findings across independent studies and is particularly attractive in federated data settings due to its minimal data sharing requirements. However, its reliance on summary statistics limits its ability to recover the full statistical structure of the original data [11]. Standard meta-analysis methods do not preserve the likelihood contributions from individual datasets, leading to a loss of granularity and limited analytical flexibility. Additionally, meta-analytic methods perform poorly when regional sample sizes are small, which may yield unstable or biased estimates [12]. This is a common challenge in multi-jurisdictional health research, where sample sizes may vary considerably across regions. Another approach is federated learning which allows multiple institutions to collaboratively and iteratively train machine learning models without sharing raw data. Each institution retains its data locally, training a model on-site and then sharing only model parameters (such as gradients or weights) with a central server, where these parameters are aggregated to form a global model. This method has gained traction in healthcare due to its privacy-preserving nature, as demonstrated by Rieke et al. (2020) and Sheller et al. (2020) [13, 14], who have explored its application in medical contexts where data security is critical. While federated learning has been widely adopted for machine learning tasks such as prediction and optimisation, it is increasingly being extended to traditional statistical modelling. Recent work has demonstrated federated implementations of generalised linear regression and Cox proportional hazards regression for clinical and epidemiological research [15–18]. These advances show that federated approaches can also support hypothesis testing and estimation of treatment effects, thus broadening their applicability beyond purely predictive contexts.

This work explores the use of a federated likelihood approach to enable the analysis of health information without direct data sharing. Likelihood functions underpin models such as logistic regression, serving as the mathematical tool that determines the parameter values that best explain the observed data. Likelihood functions, therefore, carry all the information contained in the data about a specific model, while being sufficiently aggregated to ensure no personal information can “leak through.” Conceptually, likelihood functions for various jurisdictional models can be combined to yield an overall likelihood function that is mathematically identical to what would be available if the data were combined for the analysis [19]. Unlike meta-analysis, a federated likelihood approach preserves the complete probabilistic information encoded in the local data, allowing for joint estimation of parameters under richer statistical models and leading to more accurate inference. However, sharing a closed-form solution to the likelihood function isn’t practical, since such solutions typically don’t exist for complex models and must be approximated numerically. As an alternative, we investigate a brute force strategy in which regional log-likelihood matrices are aggregated, eliminating the need to reconstruct full likelihood functions.

The effectiveness of using a likelihood-based method for the interjurisdictional analysis of health data remains unknown, and it is not yet clear how this approach compares to current meta-analysis techniques. Evaluating the effectiveness of these methodologies is crucial for developing a robust framework for health data analysis that can be applied both nationally and internationally. While theoretical results suggest that combining likelihood functions across regions can produce the same outcomes as a centralised analysis, practical challenges such as computational complexity need to be explored. This work seeks to address these pressing questions, evaluating the potential of the federated likelihood approach in overcoming the challenges associated with interjurisdictional health data analysis.

The objective of this study is threefold. First, it aims to evaluate the effectiveness of the meta-analysis technique in pooling province-specific results to produce national level estimates. Second, it seeks to assess the utility of a federated likelihood approach for interjurisdictional health data analysis. Finally, this study aims to compare the performance of a federated likelihood method with the conventional meta-analysis approach. To achieve these objectives, this study uses the well-established relationship between hypertension and chronic kidney disease (CKD) among patients with diabetes as a case study – an ideal choice given the strong, well-documented association between these conditions and their public health relevance [20]. This allows for meaningful benchmarking of the analytical methods’ accuracy and reliability.

## Methods

### Case study setting and data source

This study utilised data from the Canadian Primary Care Sentinel Surveillance Network (CPCSSN), a pan-Canadian collection of electronic medical records (EMR) encompassing over 2 million primary care patients spread across 14 regional networks [21]. CPCSSN has pan-Canadian coverage of primary care practices, with participating practices situated in the majority of provinces across Canada. Since its establishment in 2008, CPCSSN continuously receives EMR data extracted by contributing networks from participating primary care practitioners. CPCSSN contains information on consenting patients’ diagnoses, procedures, laboratory tests, medication prescriptions, and referrals. As CPCSSN data is pooled at the national level, it is one of the few data sources that allows for national level data analysis while also including meaningful differences across jurisdictional boundaries rather than creating artificial boundaries as has been done by others [22]. This allows for the assessment of this novel approach by providing a basis for comparison against the actual national values.

Adult patients (18 years of age and older) with diabetes (n = 42,341) were identified using validated case definitions [23, 24]. A unique baseline visit was defined for each patient as their first visit with a participating primary care practitioner between January 1, 2014, and December 31, 2014. In keeping with using CKD and hypertension as a case study, our analyses were restricted to patients without an existing diagnosis of CKD at any time prior to baseline and those who were not marked deceased at their baseline visit.

Diagnoses of CKD were identified using eGFR. Specifically, two or more laboratory values separated by at least 90 days but not more than 1 year apart reporting an eGFR less than 60 mL/min/1.73 m^2^, regardless of age as well as two or more laboratory values separated by at least 90 days but not more than 1 year apart reporting an eGFR less than 75, 60, and 45 mL/min/1.73 m^2^ for ages younger than 40, 40 to 64, and 65 years or older, respectively [25].

The variables examined in this study include hypertension, sex, and age. Hypertension is a dichotomous variable based on any diagnosis prior to baseline [23]. Sex is a dichotomous variable based on what was recorded in the EMR. Age is treated as a continuous variable ranging from 18 to 104, without any transformations. Age was calculated as baseline visit year minus birthyear.

### Statistical analysis

All analyses were conducted using R version 4.4.1.

Five logistic regression models were fit separately for British Columbia (n = 1,700), Alberta (n = 8,002), Manitoba (n = 6,089), Ontario (n = 23,946), and the Maritimes (n = 2,604) which includes patients from Newfoundland and Labrador, New Brunswick, Nova Scotia, and Prince Edward Island. These regions were aggregated due to sample size. Additionally, a global logistic regression model was fit which encompasses all individual patient data across the provinces (n = 42,341). In each model, hypertension serves as the primary exposure variable, while sex and age are included as covariates to control for potential confounding effects. The province of Quebec has been excluded from the analyses as there were no recorded patients with the outcome of interest. The province of Saskatchewan and the Canadian territories are not included as CPCSSN does not currently collect data in those regions.

### Meta-analysis

Common effect meta-analysis was employed to pool the coefficients from the five regional models using the R package ‘meta’. A separate meta-analysis was conducted for each model parameter to synthesise the provincial estimates of CKD across Canada. The inverse variance method was used for pooling [26]. The heterogeneity across regions was assessed using Cochran’s Q, which is a chi-square statistic comparing the effect size from each regional model to the overall pooled effect. Regional heterogeneity was measured using I^2^, which is the proportion of total variation in effects due to true heterogeneity rather than chance.

### Assumptions of the federated likelihood approach

The federated likelihood method is built upon a core set of assumptions that support both the validity and interpretability of inferences drawn from distributed data sources. These assumptions are critical for ensuring the reliability and applicability of the method, and are outlined as follows:

1. Common Effect Assumption:

A central assumption is that of a common effect. The fundamental objective of this method is to estimate a global parameter that is common across all sites. If true heterogeneity is present, more flexible models such as mixed-effects approaches may be warranted which is beyond the scope of this study.

2. Scientific Pooling Assumption (Justifiability of Pooled Analysis):

The primary assumption is that data from all participating jurisdictions or institutions can be justifiably pooled for unified analysis. This requires that the research question, study populations, and measured variables are sufficiently comparable to allow for meaningful inference. This assumption should be critically assessed a priori: if conceptual alignment across jurisdictions is lacking, then pooling – no matter how technically feasible – is inappropriate.

3. Consistency of Model Structure Across Sites:

To construct a valid joint likelihood, all participating sites must use an identical model structure. This includes consistent specification of the outcome variable, covariates, link function, and distributional assumptions. Structural inconsistencies, such as differences in variable inclusion, outcome definitions, missingness, or data transformations, would undermine compatibility and compromise the validity of joint inference.

4. Independence of Observations Across Jurisdictions (IID Assumption):

Finally, the method assumes that observations are independent and identically distributed (IID) across jurisdictions, with no overlapping or duplicated records. This condition is crucial to avoid double-counting, which can bias the likelihood.

These assumptions form the theoretical backbone of the federated likelihood framework. In practice, each must be carefully assessed within the specific context of the research question and available data. When any assumption is violated, alternative methods, such as mixed-effects modelling or meta-analytic approaches, may offer more suitable solutions.

### Federated likelihood method

As it is often impractical to share a closed-form solution to the likelihood function since such solutions typically don’t exist for complex models and must be approximated numerically, we investigate a brute force approach in which regional log-likelihood matrices are constructed and aggregated, eliminating the need to reconstruct full likelihood functions. This brute force approach utilises a grid search strategy to calculate the log-likelihood at every combination of plausible parameter values thereby constructing a matrix of log-likelihood values.

A predefined range of possible parameter values is created for each parameter in the model. These parameter values are drawn from the 95% confidence intervals associated with each of the estimates from the regional multivariable logistic regression models. Using confidence intervals to define this range is beneficial because they reflect the variability in our estimates, allowing us to incorporate regional variation into the grid search for selecting parameter values in our fully combined model. To construct the range of values for a given parameter, the smallest (most conservative) lower bound and the largest (most conservative) upper bound are identified across each of the regional confidence intervals for that parameter. These two values – representing the lowest and highest plausible values of the parameter across all models – form the endpoints of the parameter space for that vector. Once this inclusive range is established, it is segmented by generating a sequence of values at small, uniform increments. This study will explore the performance across two vector increments: 0.01 and 0.005. This results in a finely resolved vector that effectively captures the full extent of the parameter uncertainty across models. This approach ensures that no plausible values are excluded from the grid search and allows for the construction of a combined model that fully reflects the range of estimates derived from the ensemble of individual models.

Within each region, the log-likelihood was then calculated for each unique combination of values from the parameter vectors. The result of this process is a matrix of log-likelihood values, with each element of the matrix representing the likelihood of the data given a specific combination of parameter values. Technically, this will be a tensor for more than two parameters, but for simplicity we will refer to this as a matrix. This matrix effectively maps out the likelihood landscape for the regional model, allowing for a detailed examination of how the likelihood changes across different parameter combinations. By calculating the log-likelihood across all potential sets of parameter values, each matrix includes the "true" maximum log-likelihood, corresponding to the set of parameter values that best fit the regional data.

**Figure 1:**
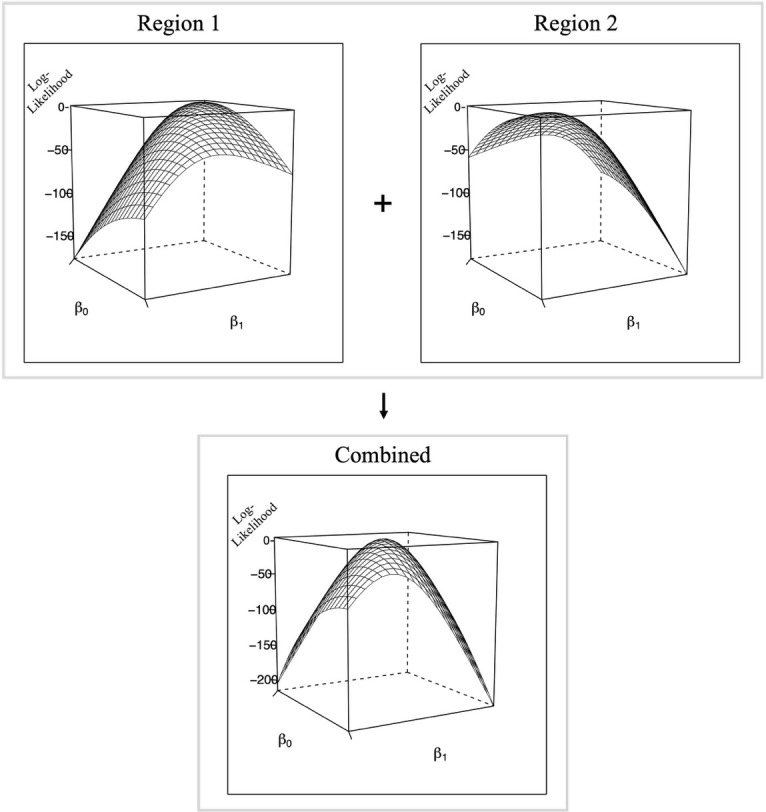
Contour plots of simulated data demonstrating the application of the likelihood method for the analysis of data across two distinct regions with two parameters

This process was then repeated independently for each regional model, generating a matrix of log-likelihood values for each region. The regional log-likelihood matrices were then summed. Summing the log-likelihoods at each intersection of parameter values leverages the additive property of log-likelihoods in independent models, allowing for the combination of information from the regions without directly accessing their raw data. The combined log-likelihood matrix represents the overall likelihood for the global model, effectively synthesising the regional information into a single estimate. An illustration of this process using simulated data is presented in Figure 1.

In addition, the 14.7% likelihood region was identified, representing the set of parameter values where the likelihood is at least 14.7% of the maximum likelihood. From this region, a 14.7% likelihood interval for each parameter can be derived, defining the range of parameter values around the maximum where the likelihood of the data is highest. The 14.7% cutoff is chosen because it approximately corresponds to the likelihood threshold associated with a 95% confidence interval under likelihood theory [27]. We report both likelihood and confidence intervals.

The parameter values that maximised the combined likelihood were identified and compared to the global model estimates to evaluate the performance of the likelihood-based approach. In addition, the estimates obtained through the likelihood method were directly compared to those generated by meta-analysis. The meta-analysis, which aggregates odds ratios across regions, and the likelihood-based method, which combines regional log-likelihoods, represent two distinct strategies for synthesising regional data into combined estimates. To assess the accuracy of each approach, the common effect meta-analysis estimates, along with the federated likelihood estimates, were compared to the global model by calculating the relative percent absolute bias (RPAB). This was defined as the absolute difference between each pooled estimate and the global estimate, divided by the global estimate. Comparing the RPABs across methods provides a measure of how closely each approach approximates the true global model and highlights potential limitations or biases inherent in either the meta-analysis or federated likelihood method.

## Results

A total of 42,341 participants were included in the analysis. The mean age of patients at baseline was 63.38 (SD: 12.22). Among the overall study population, 53% of patients were male and 47% were female. 51% of the study population has hypertension, with Ontario having the lowest proportion at 44% and the Maritimes having the highest proportion of hypertension at 67%. 15% of the overall study population have chronic kidney disease, with Alberta having the lowest proportion at 13% and the Maritimes having the largest proportion at 19%. Among the study population, 4% live in British Columbia, 19% live in Alberta, 14% live in Manitoba, 55% live in Ontario, and 6% live in the Maritimes (Table 1).

**Table 1:** Descriptive statistics of adult patients with diabetes in Canada

	***Global (N=42,341)***	***British Columbia (N=1,700)***	***Alberta (N=8,002)***	***Manitoba (N=6,089)***	***Ontario (N=23,946)***	***Maritimes (N=2,604)***
*Age, mean (SD)*	63.38 (12.22)	63.14 (12.39)	62.66 (12.11)	63.03 (12.60)	63.58 (12.09)	64.72 (12.08)
*Age range*	18–104	20–100	18–98	24–100	18–104	20–101
*Sex*						
*Male, n (%)*	22,478 (53%)	911 (54%)	4,315 (54%)	3,320 (55%)	12,606 (53%)	1,326 (51%)
*Female, n (%)*	19,863 (47%)	789 (46%)	3,687 (46%)	2,769 (45%)	11,340 (47%)	1,278 (49%)
*Hypertension*						
*No, n (%)*	20,828 (49%)	700 (41%)	3,050 (38%)	2,916 (48%)	13,292 (56%)	870 (33%)
*Yes, n (%)*	21,513 (51%)	1,000 (59%)	4,952 (62%)	3,173 (52%)	10,654 (44%)	1,734 (67%)
*CKD*						
*No, n (%)*	36,193 (85%)	1,454 (86%)	6,987 (87%)	5,235 (86%)	20,396 (85%)	2,121 (81%)
*Yes, n (%)*	6,148 (15%)	246 (14%)	1,015 (13%)	854 (14%)	3,550 (15%)	483 (19%)

*CKD=chronic kidney disease. Three missing values for sex were imputed using K-Nearest Neighbours (KNN).

We evaluated the performance of meta-analysis and a federated likelihood method for estimating the association between hypertension and chronic kidney disease across Canadian provinces. Analyses focused on assessing regional heterogeneity and estimating model accuracy compared to the true global logistic regression model. Estimates from the global and regional models, and results for each method are summarised below.

Across the five provinces studied, model estimates showed both similarities and important regional differences. The effect of hypertension on the estimated log-odds of CKD, common across all ages and both sexes, varied across regions, with the strongest associations observed in British Columbia (0.57) and the Maritimes (0.47), and a noticeably smaller effect size in Ontario (0.16).

### Meta-analysis results

**Figure 2:**
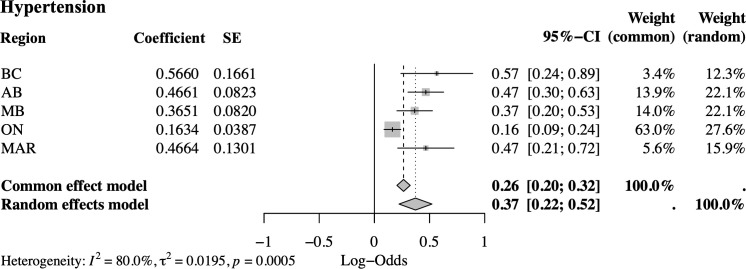
Meta-analysis results for the coefficient for hypertension, synthesising model results from British Columbia (BC), Alberta (AB), Manitoba (MB), Ontario (ON), and Maritimes (MAR)

A common effect meta-analysis was conducted to pool the coefficients from the five regional models. The regional coefficients for hypertension, and their standard errors (shown in Table 2) were used for the meta-analysis. The results are summarised in the forest plot presented in Figure 2.

**Table 2:** Global and regional logistic regression results

	***Global model***	***British Columbia***	***Alberta***	***Manitoba***	***Ontario***	***Maritimes***
	Coefficients (SE) 95% confidence intervals					
*Intercept*	–7.0460 (0.1007)	–7.0588 (0.5060)	–7.2386 (0.2456)	–6.6667 (0.2581)	–7.0716 (0.1338)	–7.1802 (0.3915)
	–7.24, –6.85	–8.08, –6.09	–7.73, –6.76	–7.18, –6.17	–7.34, –6.81	–7.96, –6.43
						
*Hypertension*	0.2542 (0.0300)	0.5660 (0.1661)	0.4661 (0.0823)	0.3651 (0.0820)	0.1634 (0.0387)	0.4664 (0.1302)
	0.20, 0.31	0.25, 0.90	0.31, 0.63	0.21, 0.53	0.09, 0.24	0.21, 0.73
						
*Sex*	–0.0737 (0.0291)	–0.1012 (0.1462)	–0.0442 (0.0708)	–0.2778 (0.0780)	–0.0034 (0.0384)	–0.2726 (0.1084)
	–0.13, –0.02	–0.39, 0.19	–0.18, 0.09	–0.43, –0.12	–0.08, 0.07	–0.49, –0.06
						
*Age*	0.0770 (0.0014)	0.0740 (0.0070)	0.0753 (0.0034)	0.0714 (0.0036)	0.0780 (0.0019)	0.0807 (0.0054)
	0.07, 0.08	0.06, 0.09	0.07, 0.08	0.06, 0.08	0.07, 0.08	0.07, 0.09

The meta-analysis for hypertension produced pooled estimates that were compared to the known global model estimate of 0.25 (95% CI: 0.20 to 0.31). The common effect meta-analysis yielded an estimated log-odds ratio of 0.26 (95% CI: 0.20 to 0.32), which closely approximated the global estimate and included it within its confidence interval. However, substantial heterogeneity was detected across regions (I^2^ = 80%, p = 0.0005), suggesting meaningful variation in the association between hypertension and CKD across regions. This level of heterogeneity suggests that the assumption of a common effect may not hold. In contexts where effect heterogeneity is substantial, estimates may be biased or oversimplified because they have been pooled across jurisdictions without adequately accounting for true between-site variation, therefore alternative approaches such as mixed-effects or random-effects models could be more appropriate. Nonetheless, the primary aim of this study was to evaluate the performance of the federated likelihood method, rather than to identify the most suitable model for inference. As such, the use of a common effect model remains justifiable within the scope of our objectives.

### Likelihood method results

**Figure 3:**
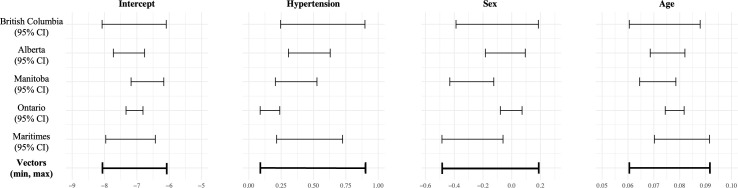
Vectors of possible parameter values. Vectors increased from the minimum value by increments of either 0.01 or 0.005 up to the maximum value

A wide parameter space was generated based on the confidence intervals from the regional multivariable logistic regression models estimating the association between hypertension and CKD. The development of the parameter vectors is illustrated in Figure 3.

Within each region, the log-likelihood was calculated for every unique combination of values from the parameter vectors, resulting in 4,438,496 evaluations per region given the threshold of 0.01, and 61,151,895 evaluations per region given the threshold of 0.005. This process produced a matrix of log-likelihood values for each regional model, effectively mapping the likelihood landscape and enabling a detailed examination of how the likelihood varies across different parameter combinations. Each matrix includes the "true" maximum log-likelihood, corresponding to the set of parameter values that best fit the regional data. The regional log-likelihood matrices were then summed to create a combined log-likelihood matrix, which was subsequently maximised to identify the parameter values that best fit the data across regions. The results of this process are presented in Table 3.

**Table 3:** Federated likelihood method (0.01, 0.005) results compared to the global logistic regression results

			Intercept	Hypertension	Sex	Age
		Maximum log-likelihood	Coefficients			
Alberta	Regional model	–2702.45	–7.2386	0.4661	–0.0442	0.0753
	Likelihood model (0.01)	–2703.44	–6.9156	0.4776	–0.0454	0.0705
	**Likelihood model (0.005)**	**–2702.45**	**–7.2556**	**0.4676**	**–0.0454**	**0.0755**
British Columbia	Regional model	–618.57	–7.0588	0.5660	–0.1012	0.0740
	Likelihood model (0.01)	–618.70	–6.8156	0.5776	–0.1054	0.0705
	**Likelihood model (0.005)**	**–618.59**	**–7.1606**	**0.5626**	**–0.1004**	**0.0755**
Manitoba	Regional model	–2184.38	–6.6667	0.3651	–0.2778	0.0714
	Likelihood model (0.01)	–2184.42	–6.6056	0.3676	–0.2754	0.0705
	**Likelihood model (0.005)**	**–2184.41**	**–6.6006**	**0.3676**	**–0.2804**	**0.0705**
Ontario	Regional model	–8939.61	–7.0716	0.1634	–0.0034	0.0780
	Likelihood model (0.01)	–8940.51	–7.2456	0.1576	–0.0054	0.0805
	**Likelihood model (0.005)**	**–8940.50**	**–7.2456**	**0.1576**	**–0.0004**	**0.0805**
Maritimes	Regional model	–1083.57	–7.1802	0.4664	–0.2726	0.0807
	Likelihood model (0.01)	–1083.57	–7.1656	0.4676	–0.2754	0.0805
	**Likelihood model (0.005)**	**–1083.57**	**–7.1706**	**0.4676**	**–0.2704**	**0.0805**
**Global model**		**–15563.98**	**–7.0460**	**0.2542**	**–0.0737**	**0.0770**
Combined likelihood model (0.01)		–15567.06	–7.2856	0.2476	–0.0754	0.0805
**Combined likelihood model (0.005)**		**–15564.56**	**–6.9406**	**0.2576**	**–0.0754**	**0.0755**

At the regional level, the estimates obtained using the federated likelihood approach closely approximated the estimates from the regional logistic regression models. Across all regions, the differences in maximum log-likelihood values between the regional and federated likelihood models (both the 0.01 and 0.005 vector increments) were negligible – typically less than one unit – indicating strong agreement in model fit. Similarly, coefficient estimates from the federated likelihood models remained consistent with those from the regional models, suggesting that the federated estimation procedure preserved site-specific inference.

For the primary exposure of interest (hypertension), the combined federated likelihood model using the 0.01 vector increment produced an estimated log-odds ratio of 0.2476, closely approximating the global estimate of 0.2542. Using a smaller increment of 0.005 further improved alignment with the global model: the log-odds ratio increased to 0.2576, and the maximum log-likelihood (–15,564.56) approached that of the global model (–15,563.98), outperforming the 0.01 model (–15,567.06). These results suggest that tighter increments enhance estimation accuracy.

Additionally, adjustment for potential confounding was maintained across all modelling strategies. By ensuring that the same covariates (sex and age) were included across jurisdictional models, the federated likelihood method was able to control for key sources of confounding.

Overall, the likelihood method effectively estimated the true global model parameters without requiring individual-level data transfer. Although slight deviations were present, the magnitude of the discrepancies in effect size was small and did not impact the overall interpretation of the findings. These results highlight the utility of the federated likelihood approach for interjurisdictional health data analysis. Table 4 summarises all of the results previously discussed.

**Table 4:** Summary of the performance of the common effect meta-analysis and the federated likelihood method relative to the global model

	**Intercept**	**Hypertension**	**Sex**	**Age**
	**Coefficients**			
**Common effects (95% CI)**	–7.0131 (–7.2644, –6.8124)	0.2646 (0.2043, 0.3248)	–0.0721 (–0.1294, –0.0149)	0.0765 (0.0738, 0.0793)
**Likelihood 0.01 (14.7% LI)**	–7.2856 (–7.3356, –7.2356)	0.2476 (0.1876, 0.2976)	–0.0754 (–0.1254, –0.0154)	0.0805 (0.0805, 0.0805)
**Likelihood 0.005 (14.7% LI)**	–6.9406 (–6.9956, –6.8906)	0.2576 (0.2026, 0.3126)	–0.0754 (–0.1304, –0.0204)	0.0755 (0.0755, 0.0755)
**Global model (95% CI)**	**–7.0460 (–7.2444, –6.8495)**	**0.2542 (0.1955, 0.3130)**	**–0.0737 (–0.1308, –0.0166)**	**0.0770 (0.0743, 0.0798)**

*LI=likelihood interval. A 14.7% likelihood interval includes a range of parameter values where the likelihood is at least 14.7% of the maximum likelihood. The 14.7% likelihood cutoff is chosen because it approximately corresponds to the likelihood values that define a 95% confidence interval under likelihood theory.

**Figure 4:**
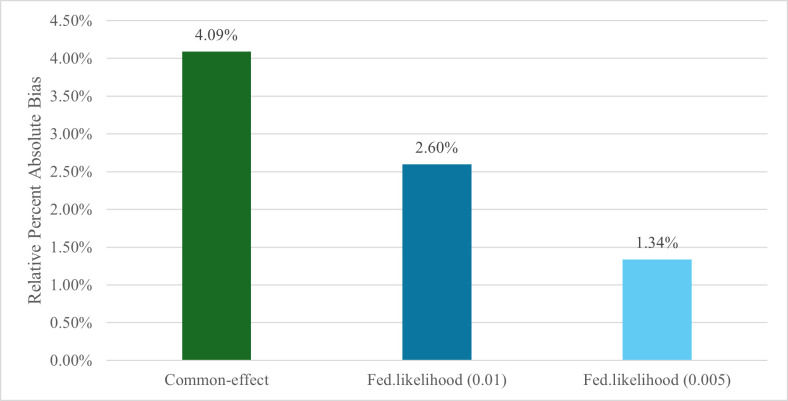
Bar graph of the relative percent absolute bias comparing the coefficients for hypertension from the common effects meta-analysis, 0.01 federated likelihood, and 0.005 federated likelihood models to the global model

The common effect meta-analysis estimates, along with the federated likelihood estimates, were compared to the global model to evaluate their accuracy. Relative percent absolute bias (RPAB), defined as the absolute difference between each pooled estimate and the global estimate divided by the global estimate, was calculated to quantify the bias in each method. Figure 4 summarises the RPAB for the coefficients for hypertension across the two approaches compared to the global model. Among all methods, the federated likelihood model with the 0.005 increment achieved the lowest RPAB (1.34%), indicating the closest approximation to the global model. The model with the 0.01 increment also performed well but showed slightly higher bias (2.60%). Both federated likelihood approaches outperformed the common effects meta-analysis, which produced moderately accurate estimates (4.09% RPAB).

Overall, the federated likelihood method produced estimates that were highly consistent with those obtained from the common effects meta-analysis approach. Notably, the federated likelihood method provided the most accurate estimate for the primary effect of interest, demonstrating a smaller RPAB compared to the common effect meta-analysis. The federated likelihood method therefore offers a robust alternative that maintains both accuracy and interpretability in federated data analysis.

## Discussion

This study introduced a real-world implementation of a federated likelihood approach to data analysis and compared this method to the existing meta-analysis framework. Overall, the federated likelihood method proved to be an effective strategy for analysing distributed data across regions where individual-level data sharing is restricted.

Both the common effect meta-analysis and the federated likelihood method produced similar results across parameters, suggesting that either approach is generally appropriate for federated health data analysis. However, the federated likelihood method with a smaller increment (0.005) consistently outperformed both the 0.01 increment model and the meta-analysis model. The federated likelihood approach with a 0.005 increment produced estimates with lower relative percent absolute bias, resulting in a closer approximation to the global model. These findings indicate that applying a tighter increment enhances the accuracy of pooled estimates. However, if the degree to which the accuracy increases does not meaningfully improve inference, then it may not justify the added computational cost of using a tighter grid. Specifically, for policy-critical decisions such as drug safety studies or resource allocation, the added accuracy from the federated likelihood method may justify the added computational cost. Whereas for exploratory analyses, meta-analysis may be sufficient.

Importantly, our analysis revealed significant heterogeneity in the effect of hypertension across regions, as evidenced by a high I^2^ statistic. This observation raises important concerns regarding the assumption of a common effect, which underlies both the federated likelihood and common effect meta-analysis models. In contexts where effect heterogeneity is substantial, this assumption may be violated, leading to biased or oversimplified estimates. In such cases, alternative modelling strategies – such as a random-effects meta-analysis or mixed-effect modelling – may be more appropriate for capturing the between-region differences in effect size. Nevertheless, the goal of this study was to evaluate the performance of the federated likelihood method rather than to identify the optimal model for inference.

This study has several notable strengths. First, it introduces and evaluates a novel methodological approach for federated health data analysis. Second, the study employs a real-world case using data from the Canadian Primary Care Sentinel Surveillance Network, providing a practical demonstration of the method’s utility in a clinically relevant context. This enhances the validity of the findings and underscores the method’s potential for direct application in health research. Third, by systematically comparing the federated likelihood approach with the common effect meta-analysis, the study offers a comprehensive performance evaluation. This comparative design enables a clear assessment of the advantages and limitations of each method, which is an important contribution to the methodological literature. Finally, the study directly compares the performance of two federated likelihood vector increments (0.01 and 0.005), demonstrating that a smaller increment improves estimation accuracy. This evaluation of methodological specification provides practical insights into implementation choices that can significantly impact model performance in real-world federated settings.

A limitation of the likelihood approach is its computational intensity. Computing a single regional log-likelihood matrix required roughly 6.5 hours of run-time; across all five regions, this amounted to roughly 32.5 hours of run-time. In contrast, the meta-analysis for hypertension completed in just 0.4 seconds. The computational burden increases substantially as heterogeneity between regions grows, as sample size increases, as the increments of the parameter vectors become smaller, as the number of model parameters increases, and as the number of regions increases. Greater heterogeneity may result in wider confidence intervals across regions, expanding the range of possible parameter values and thus increasing the number of unique combinations to evaluate. Similarly, as sample size increases, we can assume variance decreases, meaning that the use of finer increments (e.g., 0.005 as in this study) would be needed in order to properly capture the variability. However, while the use of finer increments improves approximation accuracy, it also increases the number of calculations required exponentially (the 0.01 increment resulted in 4,438,496 evaluations compared to 61,151,895 evaluations using the 0.005 increment). The addition of more parameters or more regions compound this effect, making the process increasingly demanding. Researchers aiming to implement this method should ensure they have access to high-performance computing resources or cloud-based distributed systems capable of supporting large-scale parallel processing. Additionally, the development of more computationally efficient algorithms and approximations, such as adaptive grid search procedures or stochastic approximation techniques, can support the scaling of the method to larger datasets and more complex models.

Another important limitation of this work lies in the characteristics of the case study. The empirical evaluation was based on a single real-world dataset which does not fully capture the diverse contexts in which the method could be applied. Specifically, all participating regions had relatively large sample sizes and broadly similar distributions. As a result, heterogeneity in data quality or data distributions was not assessed in this study. Differences in variable distributions, such as shifts in age ranges or outcome prevalence should be explored. Moreover, future research should explore applications involving real-world datasets with rare outcomes, where the method’s performance may be especially informative. We also acknowledge that the scope of comparisons was restricted to meta-analysis, and broader evaluations including comparisons to other federated approaches could provide deeper insights into relative performance. Addressing these limitations in future studies will help strengthen the evidence for the method’s broader utility and robustness.

Future directions for this work include further validation of the likelihood method across a wider range of model types and complexities. Extending the framework to other model types requires only replacing the binomial log-likelihood function with the model-specific likelihood function associated with the outcome distribution. All other components of the federated estimation procedure carry over directly. Testing should extend to alternative model structures such as linear regression, ordinal logistic regression, Poisson regression, and non-linear models. Moreover, the method should be evaluated in settings with increased model complexity, including the incorporation of interaction terms, to fully define the scope and limitations of the approach. In addition, future work should focus on extending the federated likelihood framework to support random-effects or mixed-effects models. This would allow for more flexible modelling of between-site variability, particularly in contexts where the common effect assumption is violated and would enhance the method’s applicability to heterogeneous real-world data.

## Conclusion

The likelihood-based method shows strong potential as an innovative approach for federated health data analysis, particularly in environments where stringent privacy regulations restrict the sharing of individual-level information. By enabling the aggregation of statistical evidence across jurisdictions without the need to transfer sensitive data, this method addresses a major obstacle to large-scale, multi-jurisdictional health research. Further investigation is required to fully evaluate its versatility and limitations. Future research should assess its performance across a broader array of model types and complexity. These extensions will be critical for defining the boundaries of the method’s utility, ensuring its operational feasibility, and understanding its robustness in more realistic and heterogeneous data environments.

## Data Availability

Data is available upon request from the Canadian Primary Care Sentinel Surveillance Network (CPCSSN).

## Abbreviations

CKD:	chronic kidney disease
CPCSSN:	Canadian Primary Care Sentinel Surveillance Network
RPAB:	relative percent absolute bias
